# The effects of placental amino acid transporter gene expression and lamb development in primiparous versus multiparous Awassi ewes

**DOI:** 10.3389/fvets.2026.1907252

**Published:** 2026-08-28

**Authors:** Umar-Faruq O. Bolaji, Ahmad A. Aboragah, Riyadh S. Aljumaah, Hani H. Al-Baadani, Maged A. Al-Garadi, Isiaka O. Olarinre, Mohammed A. Al-Badwi, Hani A. Ba-Awadh, Ibrahim A. Alhidary, Abdulrahman S. Alharthi

**Affiliations:** Department of Animal Production, College of Food and Agriculture Science, King Saud University, Riyadh, Saudi Arabia

**Keywords:** amino acid, Awassi sheep, cotyledon, parity, transporters

## Abstract

Optimal fetal growth in small ruminants fundamentally depends on the active transport of nutrients across the maternal-fetal interface. Although macroscopic variations in placental dynamics are known, the molecular mechanisms linking maternal reproductive status to placental nutrient transport pathways remain poorly defined. This study examined the influence of maternal parity on lamb birth metrics, neonatal morphological traits, serum amino acid concentrations, and the relative expression profiles of amino acid transporter genes in the cotyledons of indigenous Awassi sheep. Forty-two pregnant Awassi ewes were assigned to two experimental groups: mature young adult primiparous (*n* = 21; 20.4 ± 0.8 months of age; 1st gestation) and multiparous (*n* = 21; 38.2 ± 1.2 months of age; 2nd or 3rd gestation). Following natural singleton deliveries, phenotypic measurements and blood samples were collected from a representative cohort of ewes and their offspring (*n* = 14 pairs per parity). Total hydrolyzed serum amino acid profiles were quantified using gas chromatography–mass spectrometry. Relative mRNA expression levels of five specialized transporter genes in harvested cotyledon tissues were evaluated by quantitative real-time PCR. Lambs born to multiparous ewes had higher birth weights, wither heights, and body circumferences than those born to primiparous ewes (*p* < 0.05). Fold change in the expression of all target genes (*SLC1A5*, *SLC7A8*, *SLC38A6*, *SLC3A1*, and *SLC7A5*) in the cotyledonary tissues of multiparous ewes was increased (*p* < 0.05). Multiparous lambs had higher circulating concentrations of alanine, leucine, glycine, phenylalanine, histidine, lysine, and tyrosine, resulting in a higher total systemic amino acid concentration (*p* = 0.001). In contrast, primiparous lambs had higher concentrations of aspartic acid and glutamic acid (*p* < 0.05). In conclusion, parity-dependent regulation of cotyledonary amino acid transporters is a primary driver of prenatal resource allocation. Mature multiparous ewes upregulate essential transport machinery to clear nutrients from maternal circulation and concentrate them within the fetus, optimizing neonatal growth and structural development.

## Highlights

- Maternal parity significantly increases birth weight, wither height, and body circumference in Awassi lambs.
- Multiparous ewes show widespread transcriptional upregulation of placental amino acid transporter genes.
- Upregulated cotyledon transporters are directly correlated with higher circulating amino acid levels in newborn lambs.
- Primiparous ewes have higher maternal amino acid retention due to ongoing growth demands.

## Introduction

Awassi sheep are widely recognized for their adaptability to harsh environmental conditions and their versatile roles in milk and meat production (1, 2). A comprehensive understanding of reproductive characteristics is crucial for optimizing flock management and enhancing breeding strategies, since reproductive efficiency directly influences the sustainability of small ruminant production (3–5). Awassi sheep are widely recognized as a nonseasonal breed in most Middle Eastern countries. Unlike many temperate breeds, Awassi ewes remain reproductively active for most of the year, allowing extended breeding and lambing periods. This trait is mainly due to genetic adaptation to local environmental and climatic conditions. However, reproductive activity can still vary depending on management, nutrition, photoperiod, and location. Ali et al. (6) reported that reproductive performance is generally higher in summer and autumn than in spring and winter, likely due to these genetic and climatic factors. Sexual maturity is generally reached between 16 and 20 months of age, but this varies considerably depending on environmental conditions and nutritional status (7). In traditional management systems, conception rates range from 85 to 90%, while intensively managed flocks can achieve rates exceeding 95% (8, 9). Targeted dietary supplementation during the breeding season has also been shown to improve ovulation rates and embryo survival, potentially increasing conception rates by 15 to 20% (10–12). In addition, a study by Abdulkareem et al. (13) reported a 100% conception rate for Awassi ewes in certain regions of Iraq following a flushing and estrus synchronization regimen.

While the significant influence of maternal dietary status on fetal growth, neonatal birth weight, and subsequent offspring performance is well established (14), recent studies have increasingly focused on the physiological effects of maternal parity dynamics (15–17). Parity, defined as the number of successful pregnancies, is a primary driver of reproductive efficiency and interacts closely with maternal age, metabolic state, and seasonal variations. In small ruminants, reproductive performance often improves progressively up to the fourth parity before gradually declining (18). In contrast, primiparous ewes during their first gestation frequently exhibit lower fertility, reduced lamb birth weights, and a higher incidence of embryonic loss compared to multiparous ewes (15).

During gestation, the placenta serves as the essential structural and functional interface enabling metabolic exchange between maternal and fetal circulations. This complex organ undergoes extensive anatomical and physiological adaptations, which are strongly influenced by parity status, particularly regarding cotyledonary development (19). These parity-dependent changes fundamentally determine nutrient transfer capacity, fetal growth patterns, and overall reproductive success (20). Previous morphological studies show that multiparous ewes generally develop a more structurally efficient placenta, sometimes exhibiting a 15 to 20% increase in total cotyledon number or mass compared to primiparous ewes (21). This improved cotyledonary structure is directly associated with greater uterine capacity, more effective nutrient exchange, and increased fetal growth potential (22).

In addition to altering prenatal pathways, maternal parity status significantly affects postpartum recovery and subsequent inter-lambing intervals (23). Vasquez-Hidalgo et al. (24) showed that parity status and peri-parturient nutrient availability interactively modulate critical postpartum outcomes, including uterine involution and placental expulsion. Their findings indicate that multiparous ewes experience faster uterine recovery and a reduced risk of complications, such as retained placenta, compared to first-parity ewes.

Although existing literature documents the macroscopic effects of parity on placental morphology, birth weight, and overall lamb development, the underlying molecular mechanisms remain poorly defined. Specifically, there are limited data on how maternal parity influences the expression profiles of essential nutrient transporters within the functional units of the sheep placenta. Quantifying these molecular variations is crucial for developing precision nutritional and reproductive management strategies tailored to the physiological demands of this high-value indigenous breed. Therefore, this study aimed to investigate the influence of maternal parity (primiparous vs. multiparous) on Awassi lamb birth weight, selected neonatal morphological traits, and the relative expression profiles of key amino acid transporter genes in placental cotyledons.

## Materials and methods

### Ethical statement and study site

All experimental protocols and animal handling procedures in this study were reviewed and explicitly approved by the Ethics Subcommittee of the Research Ethics Committee at King Saud University (Approval No. KSU-SE-23-108). The trial was conducted at the Agricultural Research and Experimental Station, Department of Animal Production, College of Food and Agriculture Sciences, King Saud University, Riyadh, Saudi Arabia. All institutional and national guidelines for the care and use of laboratory and agricultural animals were strictly followed throughout the experimental period.

### Experimental animals and design

In this study, 42 pregnant Awassi ewes with a uniform baseline body condition score (BCS: 3.0 ± 0.1) were assigned to two maternal parity treatment groups: a primiparous group (*n* = 21; age = 20.4 ± 0.8 months at breeding; 1st gestation) and a multiparous group (*n* = 21; age = 38.2 ± 1.2 months at breeding; 2nd or 3rd gestation). Primiparous ewes were intentionally selected as structurally mature young adults (>18 months of age at breeding) that had completed their primary somatic frame growth prior to mating. This design minimized the confounding effects of maternal-fetal nutrient competition typically observed in growing, pubertal ewe lambs.

Using a completely randomized design, ewes within each parity group were allocated to 14 outdoor group pens (3 m^2^ per pen, 3 ewes per pen), resulting in 7 replicate pens per parity treatment group. They were maintained in these designated pens for the entirety of pregnancy, from pregnancy confirmation at day 35 until parturition. All ewes were managed under identical environmental conditions and received a pelleted complete basal diet formulated to meet National Research Council (25) requirements for late-gestation sheep (Table 1). Individual maternal body weights (BW) and BCS (1–5 scale) were recorded at baseline (day 0), on day 100 of gestation, and immediately postpartum. Daily feed allowances per pen were adjusted on day 100 of gestation based on average pen body weights to accommodate late-gestational metabolic demands, and rations were offered twice daily (08:00 and 16:00 h), targeting a 5–10% refusal margin to ensure ad libitum access. Fresh drinking water was provided ad libitum.

**Table 1:** Ingredients and chemical composition of the basal diet used in the experiment.

Ingredients (% of dietary dry matter)	Content
Barley	18.00
Wheat	29.92
Palm Kernel Meal	20.00
Soybean hulls	12.03
Wheat bran	3.00
Alfalfa	6.00
Salt	0.47
Limestone	2.58
Molasses	7.85
Commercial premix^1^	0.15
Chemical composition (as dry matter basis)	
Metabolizable Energy^2^ (ME, kcal/kg)	2,700
Dry Matter (DM, %)	92.43
Ash (%)	7.79
Organic Matter (OM, %)	92.21
Crude Protein (CP, %)	13.04
Ether Extract (EE, %)	3.70
Crude Fiber (CF, %)	13.14
Neutral detergent Fiber (NDF, %)	41.95
Acid detergent Fiber (ADF, %)	23.45

1 The basal diet was supplied as a complete pelleted diet (WAFI, ARASCO, Riyadh, Saudi Arabia).

2 Contained per kg, 10000 IU vitamin A, 1000 IU vitamin D, 20 IU vitamin E, 300 mg Mg, 24 mg Cu, 0.6 mg Co, 1.2 mg I, 60 mg Mn, 0.3 mg Se, 60 mg Zn.

To eliminate the confounding physiological impact of litter size (singles vs. twins) on placental morphology and nutrient transport kinetics, downstream tissue, serum amino acid, and neonatal morphometric analyses were restricted to ewes delivering singletons (*n* = 28 total; *n* = 14 primiparous and *n* = 14 multiparous, representing 2 single-bearing ewes per replicate pen across 7 pens per group). Ewes delivering twin offspring were excluded from molecular and biochemical profiling.

### Maternal and neonatal morphological measurements

To ensure physiological uniformity and eliminate the confounding effects of litter size on placental kinetics, a representative cohort of 28 ewes that naturally delivered singletons (*n* = 14 from the primiparous group and *n* = 14 from the multiparous group) and their corresponding single lambs were selected for phenotypic and tissue profiling. Immediately after parturition, maternal body weight was recorded for the selected ewes (*n* = 28). At the same time, neonatal birth weight was recorded for the 28 single lambs (*n* = 14 per parity group) immediately after delivery and strictly before colostrum ingestion to prevent interference from milk-derived macromolecules. To assess structural skeletal development, specific neonatal conformation traits were measured following the specialized methodology described by Obeidat et al. (26). These measurements included wither height (measured vertically from the hard floor surface to the highest point of the scapular ridge using a calibrated zoometric stick) and heart girth (measured as the thoracic circumference immediately posterior to the forelimbs using a flexible fiberglass measuring tape). These morphological metrics were collected to evaluate their linear correlations with lamb birth weight and maternal postpartum body dynamics.

### Blood and cotyledon sampling

For amino acid profiling, maternal blood samples (10 mL; *n* = 14) were collected via jugular venipuncture immediately postpartum (within 30 min of delivery, prior to morning feed access, and after an overnight post absorptive period of approximately 14–16 h following the last feeding). Neonatal blood samples (5 mL; *n* = 14) were collected concurrently from the corresponding single lambs immediately after delivery, strictly before any colostrum or milk ingestion to prevent interference from exogenously derived amino acids and colostral proteins. Blood samples were drawn into vacuum tubes containing a gel separator and clot activator, allowed to clot at room temperature for 30 min, and centrifuged at 3,000 × g for 15 min at 4 °C to obtain serum. Serum aliquots were immediately stored at −80 °C for subsequent GC–MS amino acid profiling.

Placental tissue collection was performed immediately following the natural expulsion of the fetal membranes. To standardize sample origin and minimize spatial confounding, exactly three prominent, macroscopically normal, medium-weight cotyledons (ranging from 2.1 to 4.0 g) were excised from the central zone of the chorioallantois of each placenta (*n* = 14 placentas per parity group; 2 ewes per pen). Placentas showing gross pathological signs, mechanical tearing, or delayed expulsion exceeding 4 h were excluded from sampling. From the core region of each selected cotyledon, a tissue block of approximately 100 mg was dissected using sterile scalpels. The collected tissue was gently rinsed with ice-cold sterile physiological saline (0.9% NaCl) to thoroughly remove residual maternal and fetal blood, then carefully blotted dry. For each ewe, tissue segments from the three harvested cotyledons were pooled into a single composite sample to represent that biological replicate, minimizing intra-organ expression variation. The composite samples were immediately flash-frozen in liquid nitrogen and transferred to ultra-low temperature storage at −80 °C for subsequent dual-replicate relative gene expression analysis, following a protocol adapted from McCoard et al. (22).

### Extraction and analysis of serum amino acid

Quantification of the total hydrolyzed serum amino acid profile was performed according to the specialized chromatographic methodology described by Rashaid et al. (27). To release bound residues from serum proteins, a 100-μL aliquot of ewe or lamb serum was transferred into a 4-mL glass vial and mixed with 300 μL of 6 M HCl (Darmstadt, Germany) containing the internal standard L-norvaline. The vials were hermetically sealed and incubated at 110 °C for 24 h in an aluminum heating block to complete acid hydrolysis. After cooling to ambient temperature (25 °C), the hydrolyzed samples were evaporated to dryness under a gentle, uniform stream of high-purity nitrogen gas. The remaining dry amino acid residues were reconstituted in 100 μL of analytical-grade acetonitrile. Derivatization was initiated by adding 100 μL of BSTFA (Bellefonte, PA, USA) to the mixture. The solution was sonicated for 1 min to ensure complete homogenization and then incubated at 100 °C for 30 min to complete the trimethylsilyl (TMS) derivatization reaction. The resulting TMS-amino acid derivatives were analyzed by gas chromatography–mass spectrometry (GC–MS; Agilent, CA, USA).

The GC column oven temperature program was optimized as follows: the initial temperature was held at 70 °C, increased to 170 °C at a linear rate of 10 °C/min, then ramped to 280 °C at 30 °C/min, and finally held at 280 °C for a 3-min post-run bakeout. The injection port temperature was maintained at 280 °C, and a 1 μL sample volume was introduced via an automated sampler. The mass spectrometer was operated using an electron ionization source (70 eV) in both fast-automatic-scan and selected ion monitoring modes, enabling simultaneous acquisition of total ion chromatograms and highly sensitive ion fragment data. Analytical-grade amino acid standards, including alanine (Ala), leucine (Leu), proline (Pro), isoleucine (Ile), valine (Val), cysteine (Cys), serine (Ser), threonine (Thr), glycine (Gly), methionine (Met), aspartic acid (Asp), glutamic acid (Glu), phenylalanine (Phe), histidine (His), lysine (Lys), and tyrosine (Tyr), were purchased from Sigma-Aldrich (St. Louis, MO, USA). Target amino acids were quantified based on specific retention times relative to the L-norvaline internal standard. Linear calibration curves were constructed using serial dilutions of combined reference standards across a concentration range of 0.001 to 0.3 μmol/mL, with all targeted analytes exhibiting excellent linearity (*R*^2^ ≥ 0.992; Supplementary Table S1). The limits of detection and quantification for each targeted amino acid analyte were calculated and validated according to the quality control criteria defined by Rashaid et al. (27).

### Total RNA isolation and cDNA synthesis

Total RNA was extracted from composite frozen cotyledon tissue samples using the commercial miRNeasy Mini Kit (Qiagen, Hilden, Germany) in strict accordance with the manufacturer’s specified optimization protocols (22). After extraction, the absolute concentration and raw purity of the isolated RNA were measured using a NanoDrop ND-1000 spectrophotometer (NanoDrop Technologies, Wilmington, DE, USA). The optical density (OD) absorbance ratios were calculated at A260/A280 and A260/A230 nm to assess extraction quality; only RNA extracts with an A260/A280 ratio between 1.8 and 2.1 were considered pure and selected for downstream transcription pathways. To initiate complementary DNA (cDNA) synthesis, the template RNA was diluted with ultra-pure nuclease-free water to a final concentration of 250 ng/μL. Reverse transcription was performed using standard institutional protocols and reagents (Applied Biosystems, Thermo Fisher Scientific, Waltham, MA, USA). Briefly, a 2-μL aliquot of the standardized RNA template (equivalent to 500 ng of total RNA) was combined with 1 μL of random hexamer primers, reverse transcriptase enzyme master mix components, and nuclease-free water to reach the final reaction volume (28). The mixture was briefly centrifuged and transferred to a calibrated thermal cycler programmed with the following sequential parameters: initial primer annealing at 25 °C for 5 min, enzymatic elongation at 37 °C for 120 min, and thermal enzyme inactivation at 85 °C for 5 min. The resulting cDNA matrices were stored at −20 °C prior to quantitative real-time PCR amplification.

### Quantitative real-time PCR (qPCR) gene expression analysis

The relative transcription profiles of five target amino acid transporter genes—solute carrier family 1 member 5 (*SLC1A5*), solute carrier family 3 member 1 (*SLC3A1*), solute carrier family 7 member 5 (*SLC7A5*), solute carrier family 7 member 8 (*SLC7A8*), and solute carrier family 38 member 6 (*SLC38A6*)—were evaluated (Table 2). These specific target genes were selected based on three predefined criteria: (1) functional coverage of the primary active placental transport systems (System ASC, System L, System A/N, and System b0,+) operating across the cotyledonary interface; (2) direct mediation of transplacental flux of essential branched-chain, basic, and neutral amino acids crucial for ovine fetal growth and skeletal development; and (3) documented sensitivity to maternal gestational conditioning, parity status, and placental perfusion dynamics in small ruminants. All gene-specific oligonucleotide primers were designed using the NCBI Primer-BLAST database. Real-time quantitative PCR amplification was performed and expressed as relative fold-change values coming in technical duplicates on a 7500 Fast Real-Time PCR System (Applied Biosystems, Foster City, CA, USA) using 96-well optical reaction plates. Each reaction mixture had a final volume of 10 μL, containing 5 μL of PowerUp™ SYBR™ Green Master Mix (Applied Biosystems), 2 μL of primers, 2 μL of nuclease-free water, and 1 μL of synthesized cDNA template. Beta-actin (*β-actin*) was used as the endogenous housekeeping reference gene to normalize target transcriptional variation. Each reaction of gene was performed in duplicate. Relative fold-change in gene expression was calculated using the comparative 2^−ΔΔCt^ method as described by Livak and Schmittgen (29), with the following equations: ΔCt = Ct (target gene) − Ct (*β-actin*); ΔΔCt = ΔCt (multiparous sample) − Average ΔCt (primiparous control group). All individual target gene transcription metrics are expressed as relative fold-change values compared to the control group (primiparous ewes), which was assigned a baseline normalized control value of 1.00.

**Table 2:** The list of primer sequences for target genes used in real-time quantitative PCR.

Genes	Primer sequence	Accession number
SLC1A5	F: GTCTGCAGCCTTCCGCTCAT R: TTCATGCCCTCAACCTCGCC	XM_027978525.2
SLC3A1	F: GGACTGGTGGCAGGCGG R: GGAAATCTTCAACAGCATGTCGG	XM_027966884.3
SLC7A5	F: TGACGCCTGTACCCTCACTG R: AGCTCCGGTTTCTGGTAGCG	XM_027977720.2
SLC7A8	F: TGCAAAGGAACCTTCCCAGG R: ATGATCCAGGCCATGACCCC	XM_012180978.4
SLC38A6	F: CCTTCTTCCTGAAGCCTACTGGG R: CCAGGAGAGCGACTATCAGCAG	XM_042253070.2
β-actin	F: CCTTCCTGGGTAGGTGTCG R: TGGCGTAGAGGTCCTTCCTG	AJ312193.1

SLC1A5: Solute Carrier Family 1 Member 5; SLC3A1: Solute Carrier Family 3 Member 1; SLC7A5: Solute Carrier Family 7 Member 5; SLC7A8: Solute Carrier Family 7 Member 8; SLC38A6: Solute Carrier Family 38 Member 6.

### Statistical analysis

A *post hoc* power analysis was performed using G*Power 3.1. Before analysis, data normality and homogeneity of variance were assessed with PROC UNIVARIATE procedures (Shapiro–Wilk and Levene’s tests, respectively). To control for confounding variation from litter size on placental nutrient transfer and fetal growth dynamics, only ewes carrying singletons were included in downstream profiling, ensuring uniform litter size across treatments. Additionally, because maternal age is biologically confounded with reproductive experience (multiparous ewes are inherently older), maternal parity was designated as the primary classification factor to capture the physiological differences between first-parity and experienced ewes.

Data on placental amino acid transporter gene expression, ewe and lamb serum amino acid concentrations, and lamb morphological metrics were analyzed by analysis of variance (ANOVA) using the General Linear Model (PROC GLM) procedure in SAS version 9.4 (30). Preliminary two-way ANOVA models evaluating lamb sex and the Parity × Sex interaction were examined; however, because of the 90% male cohort bias, statistical power to detect sex-driven interactions was inherently limited. Since sex effects were not significant (*p* > 0.50), the final model focused on the primary fixed effect of maternal parity to preserve degrees of freedom. To account for the pen-based design and avoid pseudoreplication, individual ewe-lamb pairs were nested within their respective replicate pens, which served as the true experimental unit for testing the fixed effect of parity. The statistical model used in this study is defined as follows:

Yijk=μ+Gi+Pj(i)+εijk

Where: Y*ijk* = The dependent variable measured on the *k*-th individual animal within the *j*-th pen of the *i*-th parity group; *μ* = The overall population mean; G*i* = The fixed effect of parity (*i* = primiparous vs. multiparous); P*j*(*i*) = The random nested effect of the *j*-th replicate pen within the *i*-th parity group (serving as the error term to test the fixed effect of parity). ε*ij* = The residual random error.

Significant differences between group means were determined using Tukey’s test, with statistical significance set at *p* < 0.05. Pearson correlation coefficients were calculated using PROC CORR to assess linear associations between neonatal growth data, structural conformation traits, and postpartum maternal body dynamics. All results are presented as mean values with the standard error of the mean (±SEM).

## Results

### Development of Awassi lambs

Effects of parity on Awassi lambs’ birth weight, wither height, body circumference, and ewes’ weight are presented in Table 3. In this study, lambs born to experienced multiparous ewes had significantly higher birth weight, wither height, and body circumference compared to those born to primiparous ewes (*p* < 0.05). In contrast to the clear differences observed in the offspring, maternal live body weight recorded immediately postpartum did not differ significantly between the two experimental groups. Multiparous ewes maintained a statistically similar body weight to primiparous ewes (*p* = 0.371).

**Table 3:** Effects of parity on Awassi lambs’ birth weight, wither height, body circumference, and ewes’ weight.

Parameters	Parity groups		SEM	*p*-value
	Lambs from Primiparous	Lambs from multiparous		
Lambs birth weight (kg)	4.39^b^	4.82^a^	0.131	0.035
Wither height (cm)	40.92^b^	44.25^a^	0.379	0.001
Body circumference (cm)	41.42^b^	43.10^a^	0.426	0.012
Ewes’ weight (kg)	64.21	65.79	1.198	0.371

Different lowercase letters (a, b) within the same row indicate significant differences (*p* < 0.05) between parities. SEM = Standard error of mean for parity effect.

The linear relationships among lamb birth weight, skeletal conformation traits (wither height and body circumference), and postpartum maternal body weight were evaluated using Pearson correlation coefficients, as shown in Table 4. Lamb birth weight showed moderate to strong, statistically significant positive correlations with lamb wither height (*r* = 0.498, *p* = 0.011) and body circumference (*r* = 0.451, *p* = 0.023). In contrast, the direct linear correlation between maternal postpartum body weight and lamb birth weight was not statistically significant (*r* = 0.287, *p* = 0.164). There was a strong positive correlation between wither height and body circumference (*r* = 0.501, *p* = 0.010). Furthermore, maternal postpartum body weight was strongly associated with lamb wither height (*r* = 0.514, *p* = 0.008) and lamb body circumference (*r* = 0.394, *p* = 0.050).

**Table 4:** Pearson correlation analysis among lambs’ birth weight, wither height, body circumference, and the body weight of ewes.

Parameters	Metric	Wither height	Body circumference	Ewes’ weight
Lambs birth weight	Pearson Correlation (*r*)	0.498	0.451	0.287
	*p*-value	0.011	0.023	0.164
Wither height	Pearson Correlation (*r*)	—	0.501	0.514
	*p*-value		0.010	0.008
Body circumference	Pearson Correlation (*r*)	—	—	0.394
	*p*-value			0.050

### Serum amino acid concentrations

Effect of parity on the serum amino acid concentration of Awassi ewes and lambs are presented in Table 5. Serum amino acid concentrations were measured in blood samples drawn from ewes immediately postpartum (in a pre-prandial state prior to morning feeding) and their single lambs prior to colostrum intake. Multiparous ewes showed significantly higher concentrations of total essential amino acids (EAA; *p* < 0.05) than primiparous ewes, whereas total nonessential amino acids (NEAA) did not differ significantly (*p* > 0.05). In neonatal circulation, lambs born to multiparous ewes exhibited significantly higher concentrations of both total EAA and total NEAA than lambs born to primiparous ewes (*p* < 0.05). Primiparous ewes had significantly higher (*p* < 0.05) postpartum serum concentrations of leucine, serine, aspartic acid, glutamic acid, and lysine compared to multiparous ewes. In contrast, multiparous ewes were increased (*p* < 0.05) levels of valine, phenylalanine, and histidine, resulting in a significantly higher (*p* < 0.05) total amino acids concentration than primiparous ewes. No significant parity-dependent differences (*p* > 0.05) were observed for ewes’ serum other individual amino acids. For lamb serum profiles, amino acid concentrations were strongly influenced by parity groups. Lambs born to primiparous ewes had higher (*p* < 0.05) serum concentrations of aspartic acid and glutamic acid. Conversely, lambs born to multiparous ewes had higher (*p* < 0.05) concentrations of alanine, leucine, glycine, phenylalanine, histidine, lysine, and tyrosine, resulting in a significantly higher (*p* < 0.05) total amino acid concentration than lambs born to primiparous ewes.

**Table 5:** Effect of parity on the serum amino acid concentration of Awassi ewes and lambs.

Parameters (mg/dl)	Parity groups							
	Ewe parity		SEM	*p*-value	Lambs parity		SEM	*p*-value
	Primiparous	Multiparous			Primiparous	Multiparous		
Essential amino acids (EAA)								
Met	0.41	0.23	0.07	0.080	1.03	0.56	0.16	0.060
His	3.42^b^	8.44^a^	0.20	0.001	1.59^b^	8.05^a^	0.13	0.001
Ile	0.32	1.16	0.32	0.090	0.36	0.37	0.07	0.910
Leu	8.50^a^	5.71^b^	0.69	0.020	2.22^b^	5.01^a^	0.40	0.001
Lys	0.91^a^	0.43^b^	0.14	0.040	0.54^b^	1.75^a^	0.16	0.001
Phe	1.49^b^	7.97^a^	0.75	0.001	2.78^b^	5.59^a^	0.49	0.001
Thr	20.52	16.55	2.20	0.230	15.97	22.24	2.29	0.080
Val	6.73^b^	16.22^a^	0.49	0.010	6.20	5.83	0.53	0.630
∑EAA	42.30^b^	56.71^a^	1.82	0.008	30.69^b^	49.40^a^	2.65	0.001
Non-essential amino acids (NEAA)								
Ala	1.95	3.26	0.61	0.160	0.27^b^	2.73^a^	0.04	0.001
Asp	0.94^a^	0.25^b^	0.06	0.001	1.80^a^	0.54^b^	0.27	0.001
Cys	5.78	5.90	0.67	0.900	3.42	3.70	0.79	0.800
Glu	1.39^a^	0.76^b^	0.09	0.001	3.25^a^	1.23^b^	0.62	0.040
Gly	15.74	18.60	2.22	0.380	5.04^b^	23.92^a^	2.00	0.001
Pro	0.56	0.87	0.16	0.200	0.59	0.52	0.09	0.590
Ser	15.45^a^	6.15^b^	0.48	0.001	12.44	13.82	0.82	0.260
Tyr	5.08	5.77	0.61	0.440	4.24^b^	7.81^a^	0.83	0.010
∑NEAA	46.89	41.56	2.14	0.410	31.05^b^	54.27^a^	3.10	0.004
Total AA	89.19^b^	98.27^a^	2.35	0.020	61.74^b^	103.67^a^	5.11	0.001

Amino acids: Met = methionine; His = histidine; Ile = isoleucine; Leu = leucine; Lys = lysine; Phe = phenylalanine; Thr = threonine; Val = valine; Ala = alanine; Asp = aspartic acid; Cys = cysteine; Glu = glutamic acid; Gly = glycine; Pro = proline; Ser = serine; Tyr = tyrosine. Different lowercase letters (a, b) within the same row indicate significant differences (*p* < 0.05) between parities. SEM = Standard error of mean for parity effect.

### Gene expression of amino acid transporters

**Figure 1:**
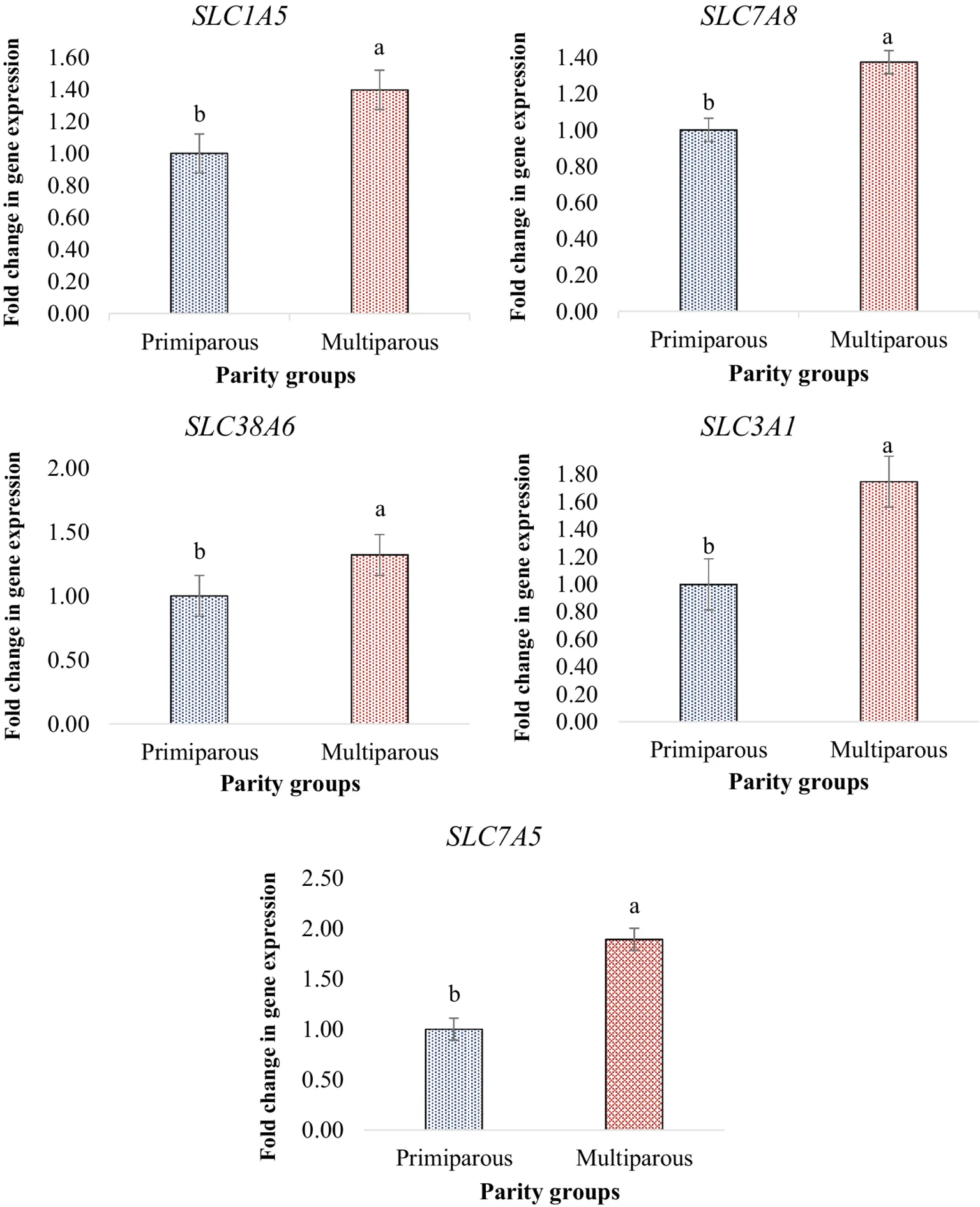
Effect of parity on amino acid transporter gene expression in the cotyledon of Awassi ewes. *SLC1A5* = solute carrier family 1 member 5 (*p* = 0.038). *SLC7A8* = solute carrier family 7 member 8 (*p* = 0.001). *SLC38A6* = solute carrier family 38 member 6 (*p* = 0.030). *SLC3A1* = solute carrier family 3 member 1 (*p* = 0.013). *SLC7A5* = solute carrier family 7 member 5 (*p* = 0.001). Different lowercase letters (a, b) within the column indicate significant differences (*p* < 0.05) between parities. SEM = standard error of mean for parity effect.

Effect of parity on amino acid transporter gene expression (*SLC1A5*, *SLC7A8*, *SLC38A6*, *SLC3A1*, and *SLC7A5*) in the functional cotyledon tissue of postpartum Awassi ewes is presented in Figure 1. Comparative real-time PCR metrics revealed that parity significantly modulates the molecular transport machinery of the functional cotyledon tissue in postpartum Awassi ewes (*p* < 0.05). The relative mRNA expression of *SLC1A5*, which mediates the transport of neutral amino acids, was higher in the cotyledons of multiparous ewes than in the baseline primiparous control group (*p* = 0.038). Similarly, the fold change in *SLC7A8* expression, responsible for maintaining amino acid balance in the placenta, was higher in multiparous ewes than in primiparous ewes (*p* = 0.001). The multiparous group also had a higher fold change in *SLC38A6* expression, which is responsible for nutrient supply in the placenta and fetal growth, than the primiparous group (*p* = 0.030). Finally, the cotyledon tissue of multiparous ewes showed an increased fold change in *SLC3A1* and *SLC7A5* expression, which are responsible for homeostasis and transport of large neutral amino acids across the placental barrier, compared to the primiparous baseline controls (*p* = 0.013 and *p* = 0.001, respectively).

## Discussion

The structural development and growth metrics of newborn lambs are vital indicators of prenatal maternal resource allocation and placental efficiency (31). The findings of this study show that maternal parity significantly affects the birth weight, wither height, and body circumference of Awassi lambs, with multiparous ewes producing offspring that are significantly heavier and structurally larger than those from primiparous ewes. These results are consistent with Kenyon et al. (32), who found that lambs born to multiparous ewes were on average 0.5 kg heavier at birth and had greater structural dimensions than those born to primiparous ewes. This phenotypic advantage is typically attributed to superior maternal nutrient reserve mobilization during late gestation, greater uterine capacity, and enhanced anatomical development of the placenta in experienced ewes (23).

Multiparous ewes generally develop a more structurally and functionally optimized placental interface, often characterized by increased cotyledon mass or total number, which significantly raises the upper limit of nutrient exchange between the maternal and fetal circulatory systems (33). Damián et al. (34) reported that lambs born to multiparous ewes typically have greater nutrient reserves, are associated with higher birth weights at parturition, and tend to have higher survival rates than those born to primiparous ewes. Additionally, colostrum and milk production in primiparous ewes during the early postpartum period are generally lower in both quality and quantity, resulting in a reduced growth rate (35). Furthermore, the moderate to strong positive correlations observed between lamb birth weight and skeletal conformation traits (wither height and body circumference) validate these parameters as reliable indicators of overall fetal skeletal development and tissue accretion. Notably, while maternal live body weight immediately postpartum did not differ significantly between parities, maternal weight was strongly associated with lamb structural dimensions. This suggests that differences in lamb development are driven by internal nutrient partitioning priorities and placental transport efficiency rather than by overall differences in maternal body size. Importantly, because the primiparous ewes in the present study were mature young adults (>18 months of age) that had completed their primary somatic frame growth, these partitioning shifts represent true parity-dependent adaptations, such as prior gestational uterine conditioning and enhanced cotyledonary transporter capacity, rather than the competition between maternal tissue accretion and fetal development that is typical of pubertal ewe lambs.

To clarify the molecular framework underlying these parity-dependent phenotypic differences, this study examined the transcriptional regulation of specific solute carrier (SLC) family amino acid transporters in functional cotyledonary tissues. The placenta acts as an active metabolic barrier, where the transport of essential and non-essential amino acids is strictly mediated by specialized transport proteins that are highly sensitive to maternal physiological status (36). Our real-time quantitative PCR data showed a marked upregulation of key amino acid transporter genes (*SLC1A5*, *SLC7A8*, *SLC38A6*, *SLC3A1*, and *SLC7A5*) in the cotyledon tissue of multiparous ewes compared to baseline primiparous ewes. This coordinated molecular increase provides a compelling explanation for the enhanced phenotypic development and altered circulating amino acid profiles observed in multiparous offspring.

The *SLC1A5* gene primarily facilitates the transport of neutral amino acids across cell membranes in mammalian systems. It has a high affinity for glutamine, alanine, serine, and cystine, and functions via a secondary active transport mechanism that utilizes the Na + gradient maintained by the Na+/K+-ATPase (37). Highly localized within the syncytiotrophoblast layer, *SLC1A5* expression is critical for sustaining cellular proliferation and fetal organogenesis by capturing precursor nutrients (38, 39). This gene is also essential for rapidly proliferating cells (40). In the present study, the significantly higher expression of *SLC1A5* in multiparous cotyledons corresponds with the broad upregulation of neutral and total cumulative amino acids delivered to the fetus. This notion is supported by recent findings from Ali and Abdulkareem (41), who observed that concentrations of both essential and non-essential amino acids declined significantly as pregnancy progressed from day 14 to day 135 of gestation, with the lowest values recorded at day 75 of pregnancy in multiparous Iraqi Awassi ewes. The reduction in maternal serum essential and non-essential amino acids was accompanied by an increase in the transfer of amino acids to the fetus via the cotyledonary interface.

This enhancement is further supported by increased expression of the L-type amino acid transporter system components, *SLC7A5* and *SLC7A8*, which specifically mediate the sodium-independent exchange of branched-chain and aromatic amino acids (42, 43). Both transporters are essential structural elements of the microvillous plasma membrane of the syncytiotrophoblast, although *SLC7A8* also functions within the basement membrane and in endothelial cells lining the fetal capillaries to regulate net nutrient flux into the fetal bloodstream (44). Our findings confirm those of McCoard et al. (22), who reported that older, more experienced ewes exhibit upregulation of *SLC7A8*, indicating increased nutrient transport capacity over successive gestations. Similarly, Erichsen et al. (45) documented the high responsiveness of the *SLC7* subfamily in cotyledonary tissues under varying nutritional conditions, highlighting its adaptability.

Crucially, this elevated L-type amino acid transporter expression functionally corresponds to the serum profiles of the offspring; lambs born to multiparous ewes showed significantly higher circulating concentrations of leucine, phenylalanine, tyrosine, and lysine. In the maternal circulation, primiparous ewes retained significantly higher levels of leucine, serine, threonine, aspartic acid, glutamic acid, and lysine postpartum. A central consideration in interpreting these findings is determining whether the observed physiological, metabolic, and transcriptional differences arise from true parity-dependent adaptations (reproductive experience and uterine tract priming) or from maternal age and somatic maturity. In adolescent ewe lamb models (<12 months of age), ongoing maternal skeletal growth actively competes with the fetus for circulating amino acids, leading to reduced placental nutrient transfer and increased maternal nutrient retention (46). In the present study, primiparous ewes were selected as mature young adults (20.4 ± 0.8 months of age at mating) that had completed primary skeletal frame growth before conception. Consequently, the enhanced cotyledonary transporter gene expression (SLC1A5, SLC7A8, SLC38A6, SLC3A1, and SLC7A5) and elevated fetal amino acid concentrations observed in multiparous ewes reflect true parity-driven physiological refinements—such as prior gestational uterine conditioning, enhanced cotyledonary vascularization, and increased microvillous membrane transporter density—rather than active nutrient diversion toward maternal skeletal growth. In contrast, the transcriptional upregulation of these key transporters suggests that the experienced multiparous placenta may function as a highly efficient conduit, potentially facilitating the clearance of these amino acids from the maternal circulation and their transport across the cotyledonary barrier to support robust fetal development (47).

A major finding of this study is the significant upregulation of *SLC38A6* expression in multiparous ewes. Members of the system A and system N transporter families, including *SLC38A6*, primarily mediate the active uptake of neutral amino acids such as glutamate and glutamine, which are essential for cellular energy metabolism and growth signaling within the placenta. Some literature suggests that SLC38A3 increased during nutrient deficiency or stress (48), as observed in the stressed placentas of primiparous ewes attempting to sustain early fetal growth. However, our results show that in Awassi sheep, *SLC38A6* expression is strongly responsive to the structural and vascular enhancements associated with multiparity. The increased cotyledonary vascularization and improved uterine perfusion characteristic of experienced ewes appear to work in tandem with upregulated *SLC38A6* activity (49, 50). This directly facilitates superior nutrient supply and corresponds to the significantly higher serum total amino acid concentrations found in both multiparous ewes and their lambs.

Furthermore, *SLC3A1* expression was significantly higher in the cotyledons of multiparous ewes. The *SLC3A1* gene encodes a heavy chain ancillary protein of the heteromeric amino acid transporter family, serving as an essential component of the b(0,+) transport system. This system regulates amino acid homeostasis during gestation by mediating the high-affinity exchange and reabsorption of dibasic (cationic) amino acids, such as histidine and lysine, along with cystine (51). In ruminants, amino acid transport and metabolism are crucial during pregnancy, when fetal nutritional requirements for growth and development are very high (52). While Gao et al. (38) reported that *SLC3A1* is weakly expressed in most non-pregnant sheep uterine cell types, its marked upregulation in the cotyledons of multiparous ewes in our study highlights its role as an adaptive molecular mechanism. Successive pregnancies impose cumulative metabolic conditioning on the ewes. The upregulation of *SLC3A1* represents a conserved physiological adaptation in ruminants, where increased transport of basic amino acids optimizes reproductive performance and offspring productivity as the ewes matures.

The close association between placental transporter gene expression and neonatal serum amino acid profiles demonstrates a functional relationship. Newborn multiparous lambs experienced a broad, systemic enrichment of both essential and non-essential amino acids, including alanine, leucine, glycine, phenylalanine, histidine, lysine, and tyrosine, resulting in an exceptionally high total amino acid concentration compared to primiparous lambs. This distinct profile supports a direct functional link between increased cotyledonary transporter transcription and greater fetal availability of the building blocks needed for tissue hypertrophy and skeletal growth (53). In contrast, primiparous lambs showed elevated concentrations of only aspartic acid and glutamic acid. These acidic amino acids are heavily utilized in transamination reactions and energy production pathways in the fetal liver and placenta (54). Their accumulation in primiparous offspring suggests an altered metabolic state, possibly due to limited availability of essential neutral and basic amino acids, which could restrict protein synthesis and contribute to lower birth weights.

A potential limitation of the present study is the skewed sex ratio of singletons (90% male), which occurred naturally across birthings. Although preliminary statistical screening showed no main effect of lamb sex within this sample, the low representation of female lambs limits the statistical power to detect subtle sexual dimorphism or parity × sex interactions. Male and female fetuses are known to exhibit distinct placental adaptations and nutrient prioritization strategies under varying maternal conditions. Consequently, the pronounced transcriptional upregulation of cotyledonary amino acid transporters observed in multiparous ewes may primarily reflect the metabolic demands of male fetal development. This sex bias should be considered when comparing these findings with studies that have balanced or female-biased cohorts, and future studies using sex-balanced or single-sex design blocks are warranted to clarify sex-specific placental dynamics. A related limitation is that, by design, maternal age and parity are confounded in this study: primiparous ewes (20.4 ± 0.8 months) were substantially younger than multiparous ewes (38.2 ± 1.2 months) at parturition. Although primiparous ewes were deliberately selected as post-somatic growth young adults rather than growing adolescent ewe lambs to minimize maternal-fetal nutrient competition, minor residual maternal growth or physiological maturation cannot be entirely ruled out. The phenotypic, biochemical, and transcriptional differences reported here should therefore be interpreted as parity-associated effects that remain inherently intertwined with maternal age. Future studies using strictly age-matched ewes across parities are needed to fully decouple the specific contributions of parity from those of maternal aging.

## Conclusion

In conclusion, variations in amino acid transporter expression in the cotyledon are influenced by structural and functional adaptations related to parity. Multiparous Awassi ewes develop a mature, efficient, and well-perfused placentome network that upregulates the transcription of *SLC1A5*, *SLC7A8*, *SLC38A6*, *SLC3A1*, and *SLC7A5*. This molecular upregulation efficiently removes key amino acids from maternal circulation and concentrates them in the fetal bloodstream. This directly explains the significantly superior neonatal growth and structural conformation metrics observed in multiparous offspring. These findings show that parity-dependent regulation of placental molecular machinery is a primary driver of fetal nutrient supply, providing valuable insights for refining precision nutritional management strategies for indigenous small ruminants throughout their reproductive lifespans.

## Data Availability

The datasets presented in this study can be found in online repositories. The names of the repository/repositories and accession number(s) can be found in the article/Supplementary material.
