# Supplementary material for: The effects of placental amino acid transporter gene expression and lamb development in primiparous versus multiparous Awassi ewes
Source: Front Vet Sci. 2026 Aug 28;13:1907252. doi: 10.3389/fvets.2026.1907252 (PMC13561948; doi:10.3389/fvets.2026.1907252)
Supplement: Supplementary file 1 [file Table_1.DOCX]

| Table S1. Linear regression parameters and calibration metrics for GC-MS serum amino acid quantification | | | | | |
| --- | --- | --- | --- | --- | --- |
| Amino Acid | **Linear Equation (y=mx+c)** | **Coefficient (R2)** | **Linear Range (μmol/mL)** | **LOD (μmol/mL)** | **LOQ (μmol/mL)** |
| Alanine (Ala) | y = 124.52x + 0.012 | 0.998 | 0.001–0.300 | 0.0003 | 0.0009 |
| Leucine (Leu) | y = 118.34x + 0.008 | 0.997 | 0.001–0.300 | 0.0004 | 0.0011 |
| Proline (Pro) | y = 95.12x + 0.015 | 0.995 | 0.001–0.300 | 0.0005 | 0.0015 |
| Isoleucine (Ile) | y = 112.80x + 0.005 | 0.996 | 0.001–0.300 | 0.0004 | 0.0012 |
| Valine (Val) | y = 130.15x + 0.010 | 0.999 | 0.001–0.300 | 0.0003 | 0.0009 |
| Cysteine (Cys) | y = 78.45x - 0.004 | 0.992 | 0.001–0.300 | 0.0008 | 0.0025 |
| Serine (Ser) | y = 104.22x + 0.011 | 0.996 | 0.001–0.300 | 0.0005 | 0.0014 |
| Threonine (Thr) | y = 98.60x + 0.007 | 0.994 | 0.001–0.300 | 0.0006 | 0.0018 |
| Glycine (Gly) | y = 142.10x + 0.018 | 0.998 | 0.001–0.300 | 0.0003 | 0.0010 |
| Methionine (Met) | y = 88.30x + 0.003 | 0.993 | 0.001–0.300 | 0.0007 | 0.0021 |
| Aspartate (Asp) | y = 108.90x + 0.009 | 0.995 | 0.001–0.300 | 0.0005 | 0.0016 |
| Glutamate (Glu) | y = 115.40x + 0.014 | 0.996 | 0.001–0.300 | 0.0004 | 0.0013 |
| Phenylalanine (Phe) | y = 121.70x + 0.006 | 0.997 | 0.001–0.300 | 0.0004 | 0.0011 |
| Histidine (His) | y = 82.15x + 0.002 | 0.993 | 0.001–0.300 | 0.0007 | 0.0022 |
| Lysine (Lys) | y = 101.50x + 0.013 | 0.995 | 0.001–0.300 | 0.0005 | 0.0015 |
| Tyrosine (Tyr) | y = 92.80x + 0.008 | 0.994 | 0.001–0.300 | 0.0006 | 0.0019 |
